# Clinical Conundrums: Differentiating Monkeypox From Similarly Presenting Infections

**DOI:** 10.7759/cureus.29929

**Published:** 2022-10-04

**Authors:** Azhar Hussain, Jasndeep Kaler, George Lau, Tyler Maxwell

**Affiliations:** 1 Pharmacology, Touro College of Pharmacy, New York City, USA; 2 Medicine, Windsor University School of Medicine, Cayon, KNA; 3 Anesthesiology, University of Massachusetts Chan Medical School-Baystate, Worcester, USA; 4 Infectious Diseases, Touro College of Pharmacy, New York City, USA

**Keywords:** disseminated rash, vesicular rash, infectious disease pathology, infection microbiology, monkeypox virus

## Abstract

Post the coronavirus disease 2019 (COVID-19) pandemic, there arises the concern of a new epidemic as cases of monkeypox are being confirmed, globally. With the initial clinical manifestation of monkeypox resembling that of the common cold or seasonal flu, recognizing alternative differential diagnoses is imperative as a medical health practitioner. The characteristic monkeypox maculopapular rash with the progression to vesicles and pustules before scabbing can be described in several other infections. Understanding the disease progression and distinct clinical presentation of monkeypox in its various stages may allow for a more expedient diagnosis among healthcare providers. Though eradicated, the clinical presentation of smallpox is the most similar to that of monkeypox; however, smallpox is no longer a concern for the general population. Other conditions such as molluscum contagiosum, syphilis, varicella zoster, measles, rickettsialpox, and scabies can present with rashes that may resemble singular or multiple states of the monkeypox rash progression. The ability to correctly diagnose an individual’s condition promptly may allow healthcare providers to provide correct supportive therapies or treatments.

## Introduction and background

With 68,017 confirmed cases in 106 different regions across the globe as of September 29, 2022, monkeypox is creating a worldwide fear of being the focus of another epidemic [1]. Monkeypox is one of the many zoonotic viruses that belong to the *Orthopoxvirus* genus of the Poxviridae family [2]. Poxviruses are large, double-stranded DNA viruses with a genomic size ranging from 130 to 360 kilobase pairs (kbp) [3]. The poxvirus family replicates in the cytoplasm of the host cell. Poxviruses will rely extensively on virus-encoded proteins that enable the viruses to replicate in the cytoplasm [2]. The central part of the genome contains genes involved in key functions such as transcription and virus assembly, whereas those located at the termini are involved in virus-host interactions [3]. Of more than 150 genes encoded by poxviruses, 49 are common to all sequenced members of this family, and 90 are common within the subfamily of chordopoxviruses [4]. The majority of these genes are conserved amongst viruses are related to viral function and form the central part of the genome [3].

Monkeypox was not recognized as a distinct human disease until 1970, when the eradication of smallpox revealed the occurrence of smallpox-like illness in rural areas [5]. Before 1970, monkeypox was identified in laboratory monkeys sent from Africa for research at the State Serum Institute in Copenhagen, Denmark [6]. Initially endemic to sub-Saharan Africa, monkeypox gained global recognition following a 2003 United States (US) outbreak. In the summer of 2003, a cluster of monkeypox cases was identified in the US Midwest, associated with pet prairie dogs. After a small rodent importation from Ghana, Africa, to Texas and further transported to Illinois, rodents were housed in close quarters to prairie dogs in a pet shop. These prairie dogs were then sold as pets before showing symptoms of infection and are thought to be the primary source of the outbreak [5]. Since 2003, several cases of monkeypox have been reported in various countries, with the largest outbreak experienced in Nigeria in 2017 [7]. An epidemiological modeling study reported the R0 value of monkeypox to be between 1.10 and 2.40 in countries where exposure to orthopoxvirus species is negligible. This elevated R0 value of monkeypox suggests widespread virus transmission may be more likely in settings where orthopoxviruses are not endemic [2,7]. Such an R0 suggests that a patient infected by monkeypox possesses the ability to infect one to two other people, and due to this heightened transmissibility, infected individuals must take special precautions to social distance and quarantine themselves [2]. The modes of transmission of monkeypox are similar to other infectious diseases.

The primary mode of transmission is direct contact with infected humans or infected animals. Animal-to-human transmission occurs through direct contact or exposure to infected animals and, most commonly, through bodily fluids such as saliva, respiratory excretions, or could exudate from cutaneous or mucosal lesions [2,8]. Human-to-human transmission has historically been reported as spread through respiratory droplets; however, contact with contaminated objects/surfaces is also deemed a risk factor for viral transmission amongst observed individuals. Monkeypox virus shares an infectious pathophysiologic response similar to smallpox, beginning with the exposure of the virus to the host [2]. The entire incubation period typically lasts 7-14 days with an upper limit of 21 days [9]. During the incubation period of monkeypox, there is no clinical presentation of the viral disease. Following the incubation period is the prodromal stage in which an individual presents with symptoms and is considered infectious. Monkeypox replicates upon initial infection and subsequently spreads to primary lymph nodes through primary viremia. From primary lymph nodes, the viral load spreads through the bloodstream, as a secondary viremia, to distal lymph nodes and organs.

**Figure 1:**
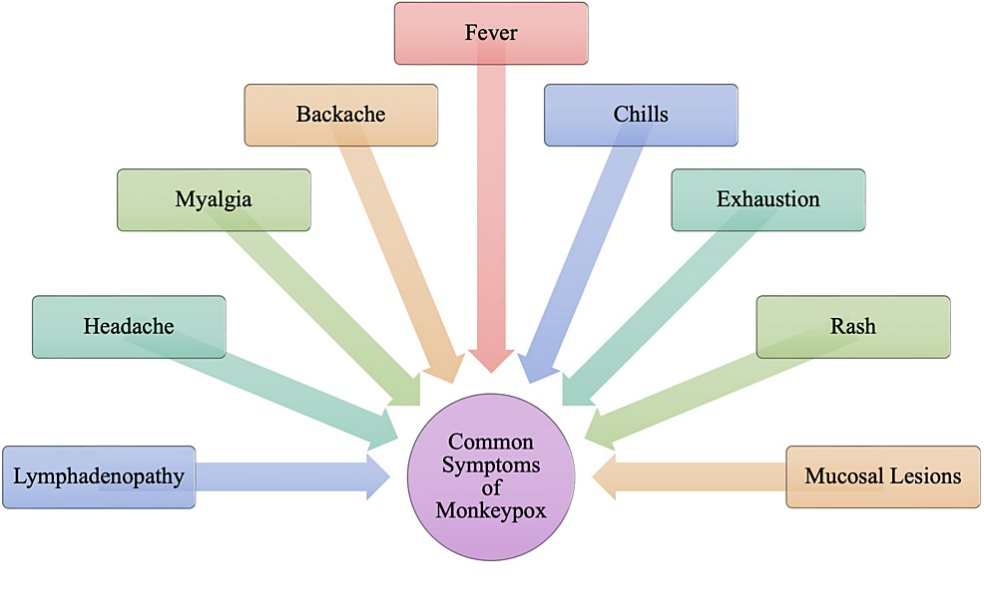
Common (Nonspecific) Symptoms of Monkeypox

Many of the initial symptoms seen in monkeypox are non-specific and can be mistaken as symptoms of a common cold or seasonal flu. Figure 1 outlines two clinical symptoms that allow for the differentiation of monkeypox from the common cold and other similar conditions. During the initial stages of monkeypox, lymphadenopathy may occur as the virus invades the lymphatic system. As the enlargement of the lymph nodes is not always associated with more common infections, this may be a differentiating factor. Lymphadenopathy will be the characteristic differentiating symptom. Following potential non-specific symptoms and lymphadenopathy by approximately one to three days, the characteristic monkeypox rash typically develops [2]. These prodromal symptoms may be mild or not present at all, suggesting that some individuals may be asymptomatic until the appearance of the rash [10]. The typical course of monkeypox consists of a fever that resolves immediately following the onset of a disseminated vesiculopustular rash or up to three days after the onset of the differential rash [11]. The characteristic rash may be found on many sections of the body. Historically, the rash has begun on the face. It then would spread in a centrifugal distribution across the body, with lesions on the extremities and face rather than the abdomen and trunk [10,11]. A centrifugal distribution of the rash means there would be lesions on the extremities and the face rather than on the abdomen and trunk. Interestingly, the 2022 outbreak of monkeypox does not always appear to follow this historical disease progression. Many patients initially notice lesion formation in both the oral cavity and the anogenital region with potential further propagation of the disease.

**Figure 2:**
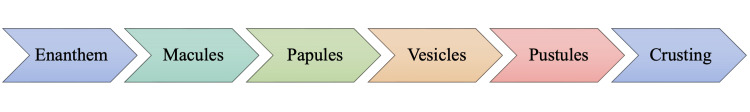
The Progression of the Rash in Monkeypox

While a rash is an important clinical symptom of monkeypox, it is a clinical feature of other conditions as well. Due to the similarity of the type of rash amongst different conditions, it is imperative to be able to differentiate these symptoms to ensure a correct diagnosis. The rash goes through various stages, with infectious lesions that may present initially as enanthem with progression to macular, papular, vesicular, and pustular lesions, as shown in Figure 2 [12]. As monkeypox progresses through many distinct types of lesions, this may increase the likelihood of misdiagnosis for rashes that may present similarly at a distinct stage and highlights the importance of having a broad differential to identify these unique diseases.

**Figure 3:**
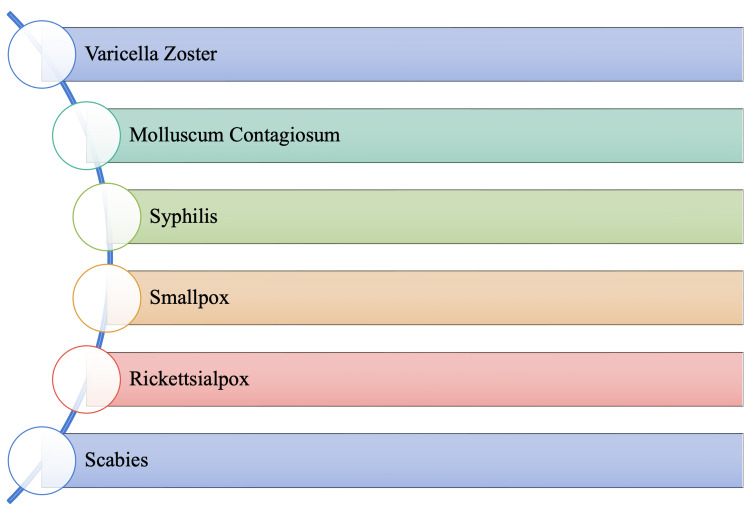
Potential Differential Diagnoses of Monkeypox

Based mainly on the clinical presentation, Figure 3 suggests alternative conditions that should be considered causative agents. Each differential diagnosis listed in Figure 3 holds clinical similarities to monkeypox and may lead to an increased likelihood of misdiagnosis. This manuscript aims to summarize the pathophysiology, modes of transmission, and clinical presentations of these similar infections.

## Review

As the incidence of monkeypox cases increases, it is imperative to educate healthcare providers on specific differential diagnostic symptoms. 

Varicella zoster virus

Varicella zoster virus (VZV) belongs to the Herpesviridae family. It is a large, enveloped virus with an icosahedral capsid and a double-stranded DNA genome just under 125,000 base pairs, containing 68 unique open reading frames (ORF) [13,14]. VZV exclusively infects humans with no animal reservoir, and the main targets are T-lymphocytes, epithelial cells, and ganglia [15].

VZV causes two distinct infections, classified as either a primary infection or a secondary infection. Varicella (chickenpox), a generalized illness, is the primary infection, and zoster (shingles) is its secondary infection caused by the reactivation of VZV from latency [16]. The primary infection of chickenpox is typically seen in children in locales where vaccination is not practiced [14]. Following the primary infection, the virus becomes latent in ganglionic neurons. No virus particles are produced during this period, and no apparent neuronal damage occurs [15]. Reactivation of VZV could occur decades later, either spontaneously or triggered by immunosuppression, trauma, infection, malignancy, etc. [14]. The incidence and severity of shingles increase with age due to declining cell-mediated immunity to VZV [17].

VZV infection is a highly contagious rash illness transmitted by inhalation of saliva droplets of subjects with an acute infection or, rarely, by direct contact with skin lesions of infected individuals [13]. Following transmission, VZV will proliferate in the oral pharynx, primarily in the tonsils and epithelial cells of the upper respiratory tract. The virus then infects the T-lymphocytes that enter circulation and constitute a primary viremia that occurs four to six days after infection [13,18]. During primary viremia, the virus is disseminated to reticuloendothelial tissues, including the liver and spleen, where it will further multiply [13]. Secondary viremia occurs when VZV is transported to the skin and mucous membranes from the reticuloendothelial tissues about 14 days after the infection [19]. Memory T-cells are believed to be crucial in promoting viral replication within epithelial cells. The viral gene products will downregulate the interferon (INF), a response mounted by adjacent epidermal cells [13].

**Figure 4:**
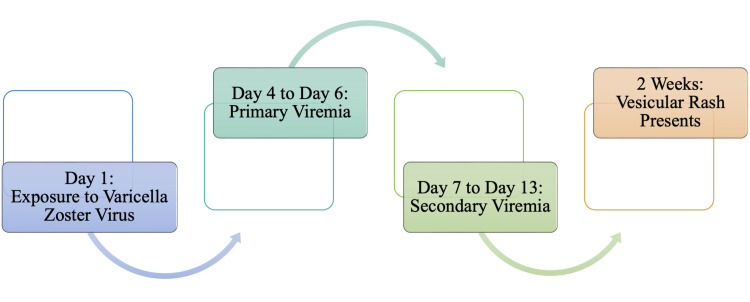
Proposed Timeline from Exposure to Varicella Zoster Virus to Presentation of Vesicular Rash

Once the antiviral response has been overcome, the viral replication in infected keratinocytes will ensue cell damage and inflammation and initiate immune responses that will cause the formation of vesicles filled with virions [13,14]. Following primary and secondary viremia, a highly contagious vesicular skin rash develops up to about two weeks following exposure to VZV, as outlined in Figure 4. It is also important to understand that an individual will be deemed highly contagious in the window presented in Figure 4: maximally contagious one to two days before the onset of a rash and during the first five to seven days after the appearance of the rash [13]. We can also observe the contagious window 12-21 days post-exposure [18]. The prodromal stage during the incubation period includes symptoms including generalized malaise, nausea, loss of appetite, high fever with a temperature up to 102°F for up to two to three days, and headache [13,18]. These prodromal symptoms are often less severe in children than in infants. Infants, adults, pregnant women, and immunocompromised people are at higher risk of severe disease and have a higher incidence of complications [18]. The clinical presentation of varicella mirrors that of monkeypox closely; however, lymphadenopathy is often noted within monkeypox infections and is absent among individuals infected with the varicella virus. The prodromal symptoms of VZVinfection can also be mistaken as the common cold or the seasonal flu due to the nonspecific symptoms that do not allow for any clear and concise diagnosis. Monkeypox is currently most commonly observed in middle-aged males, specifically those around 40 years, with a median age of 31 [2]. Some major complications that can develop in monkeypox infections include bacterial superinfection, pneumonia, and permanent scarring, which can also be served in VZV infections [2,13]. Again, these complications are of greater concern in immunosuppressed individuals.

**Figure 5:**
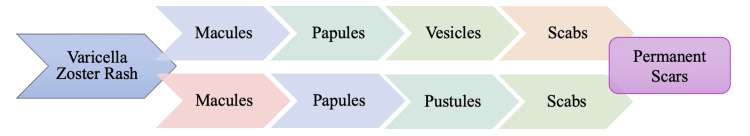
Progression of Varicella-Zoster Rash

The rash associated with VZV follows one of two progressions. Both progressions are outlined in Figure 5. The most significant difference between the two progressions of the VZV rash is whether vesicles or pustules form after the initial slight, pruritic maculopapular rash. The rash maximally involves the trunk, with small maculo-papules spreading to the necks and limbs [13,18]. After 12-72 hours, the maculopapular lesions will progress into pustules or vesicles [13]. Though both vesicles and pustules are fluid-filled blisters, the difference between the two is the type of fluid within the blister. Both vesicles and pustules are raised lesions; however, vesicles are filled with a clear fluid, whereas pustules are filled with a purulent, opaque fluid [2]. The lesions in monkeypox will first present as enanthem, lesions that develop in the mucosal membranes; however, the lesions in VZV infections appear in waves everywhere, including the mucosal membranes [2,13]. The pustules and vesicles of VZV will typically heal without any sequelae. Furthermore, any trauma to these blistered lesions can cause them to rupture and, thus, become infected by staphylococci and streptococci bacteria, leaving a permanent scar, as depicted in Figure 5 [13].

In this manner, the clinical presentation of the VZV rash is similar to the lesions observed in monkeypox. The most considerable observable difference between these two infections lies in the anatomical distribution of the lesions. In monkeypox infections, the vesiculo-pustular rash presents in a centrifugal distribution with more lesions on the extremities and the face rather than on the abdomen and trunk [2]. The pathophysiology of the zoster rash is defined by the reactivation of the VZV that became latent in the ganglionic neurons. Herpes-zoster rashes are characterized by clusters of vesicular lesions that run along a dermatome innervated by a single nerve fiber and are commonly observed on the chest [13,18]. Zoster lesions are also present with a similar progression of lesions as noted in Figure 5; however, these lesions are accompanied by localized pain that, in some individuals, may be intense enough to necessitate anesthetic administration [13]. In many cases, pain can persist for months after the rash has subsided in a phenomenon termed post-herpetic neuralgia [18]. As both varicella and zoster rashes present with differing symptomology, the varicella presentation of VZV infection seems to present more similar to that of monkeypox infection.

Molluscum contagiosum

**Figure 6:**
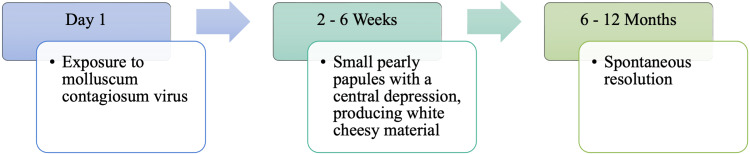
Timeline from Exposure to Molluscum Contagiosum Virus to the Presentation of Lesions

Molluscum contagiosum is a self-limited infectious dermatosis frequently seen in pediatric populations, sexually active adults, and immunocompromised individuals [20]. Like monkeypox, molluscum contagiosum is also caused by a poxvirus, specifically the molluscipox virus in the Poxviridae family [21]. The molluscum contagiosum virus (MCV) is a large brick-shaped, double-stranded DNA virus that is 200 to 300 nm in length [22]. There are four different subtypes of MCV: MCV-1, MCV-2, MCV-3, and MCV-4, with the latter being extremely rare [22,23]. MCV-1 is commonly the cause of molluscum contagiosum in children, while MCV-2 tends to be more sexual transmission and infections in older patients [22]. While molluscum contagiosum occurs globally, it is more common in endemic, densely populated communities with poor hygiene and economically disadvantaged areas [21,24]. molluscum contagiosum is a highly contagious viral infection and, as such, is very easily transmitted among individuals. Transmission of the MCV can occur through direct contact with active lesions or autoinoculation, through indirect transmission by sharing personal tools such as towels, bedding, razors, clothes, and transmission through sexual contact [24]. MCV infects the epithelial cells and replicates in the stratum spinosum layer of the epidermis and cannot be transmitted through respiratory droplets [21,25]. The virus remains in the epidermis and does not spread via the bloodstream; thus, symptoms involving other organ systems are rarely noted. Molluscum contagiosum is a self-limited infection, and the time in which the disappearance of papules varies greatly, as noted in Figure 6. During the initial two to six weeks, some papules may disappear, and new ones may appear, likely due to the autoinoculation of the virus. Individual lesions are described as small papules that are either fleshy or pale pink [24]. Lesions often appear as small, 1-2 mm “pearly” white or flesh-colored papules that are smooth and dome-shaped with central umbilication [22]. The central umbilication of the papules may not be visible in the smaller lesions. As the lesions enlarge, this feature may become more prominent with the enlargement of the umbilication in tandem with the enlargement of the lesion. In molluscum contagiosum, lesions often cluster in one to two areas, especially in skin folds such as those found in the axilla, neck, and inguinal areas [24]. The anatomical location of the lesions depends on the patient’s age. Pediatric patients often experience a more diffuse and extensive disease as compared to infected adults. Children commonly develop lesions on the face, trunk, and upper extremities with a linear distribution, indicating autoinoculation of the virus from scratching; lesions on the palms, soles, and mucous membranes are relatively rare [24,26]. In infected adults, lesions are characteristically observed on the thighs, inguinal region, buttocks, and lower abdominal wall, and less commonly on the external genitalia and perianal region [22,24]. The lesions themselves are not pruritic, though the skin around the molluscum papules becomes pink, rough, and itchy, leading to scratching and further autoinoculation [27]. The number of lesions is typically less than 20; however, numbers can increase up to the hundreds, especially in immunocompromised individuals [24]. The distribution of the MCV lesions often presents differently than lesions observed in monkeypox infection. While MCV lesions are often clustered and number less than 20, monkeypox lesions have frequently been described in the anogenital region and are more commonly associated with pain. Moreover, the lesions are much more dispersed and do not vary whether the rash is present in adults or children. When molluscum papules resolve, an individual may be left with pink-purple or white spots that diminish over time [27].

Due to monkeypox and molluscum contagiosum being from the same family, the complications of infection are similar. Monkeypox can cause a corneal infection that can lead to permanent cornea scarring, resulting in vision loss [2]. Molluscum contagiosum, on the other hand, can cause unilateral refractory conjunctivitis when it infects the eyelid; however, this is considered rare [22]. These eyelid lesions are typically the result of autoinoculation. The most frequent molluscum contagiosum complication is molluscum dermatitis, which appears 1-15 months after the onset of the lesions in up to 10% of the patients [27]. Molluscum dermatitis consists of a well-defined, eczematoid reaction 3-10 cm in diameter with approximately a single lesion, may involve only part of a lesion, and usually disappears when the lesion heals. Other complications of MCV infection include secondary bacterial infection, inflammation, and irritation, similar to those seen in monkeypox [2,21].

Syphilis

Classified as a sexually transmitted disease, syphilis is caused by *Treponema pallidum* (*T. pallidum*), a bacterium classified under the Spirochaetaceae family [28]. *T. pallidum* is a highly motile spirochete organism with tapering ends and 6 to 14 spirals [29]. *T. pallidum* is a slow-metabolizing organism with an average multiplication time of approximately 30 hours [30]. Syphilis has always been a significant public health problem. After transmission declined to a historic low in the year 2000, the number of syphilis cases in the United States has since increased and now exceeds 55,000 new cases yearly [30,31]. In 2020, there were 133,945 new cases of syphilis, with the highest incidence occurring in men who have sex with men (MSM) populations; however, cases among heterosexual individuals appear to be increasing as well [31]. Humans have been noted as the only hosts for this organism. Though monkeypox has not been classified as a sexually transmitted disease like syphilis, direct contact with lesions during close contact has raised concern for its transmission during sexual intercourse [2]. Cases of vertical transmission from mother to fetus can also occur in monkeypox and syphilis, and recent increases in congenital syphilis cases have been observed [31]. 

**Figure 7:**
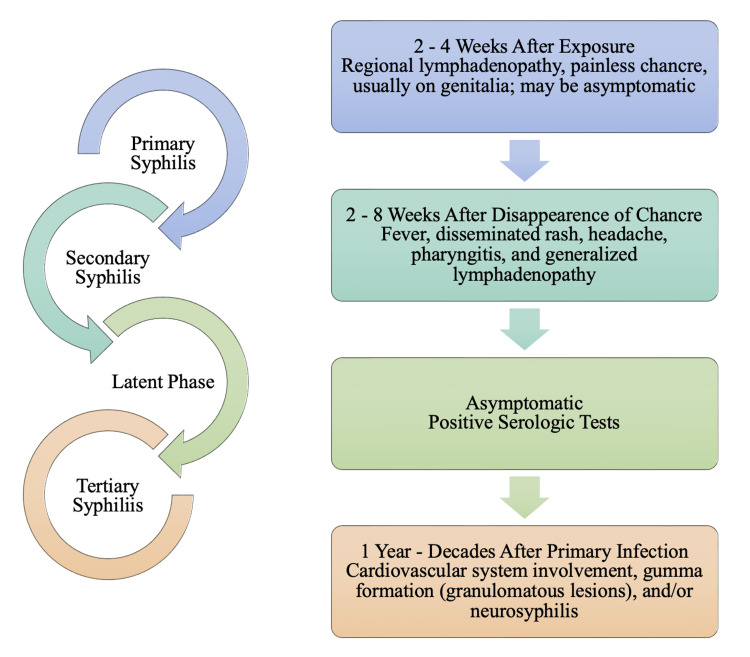
Progression of Syphilis and Clinical Manifestation of each Stage

Sexual transmission of syphilis can occur through inoculation of tiny abrasions from sexual trauma. This causes a local response, resulting in erosion and ulcer formation [32]. This characteristic ulcer of primary syphilis is referred to as a chancre, as depicted in Figure 7. This chancre is often found on the external genitalia but can develop on any site of inoculation, including the perineum, cervix, anus, rectum, lips, oropharynx, and hands [29]. Without treatment, the painless chancre will typically heal on its own within one to three weeks. Despite symptomatic resolution of the chancre, the infection may progress to secondary syphilis. Due to the nature of primary syphilis symptomology, many patients are unaware that they have been infected until the second stage of syphilis. Of the different stages of syphilis, secondary syphilis is the stage that presents with a clinical manifestation that may mimic the symptomatic presentation of monkeypox. Clinical manifestation of secondary syphilis is characterized by a rash, fever, headache, pharyngitis, and lymphadenopathy [29,32].

Along with nonspecific symptoms that are noted in secondary syphilis, systemic manifestations are also noted. An individual with secondary syphilis can also present with hepatitis, glomerulonephritis, periostitis, and early neurologic complications such as uveitis and meningitis [29]. Due to the diverse manifestations of secondary syphilis, it is commonly referred to as the ‘great imitator,’ as many other diseases can be considered in the differential diagnoses [31]. 

Without any treatment, primary and secondary syphilis manifestations will generally resolve within a few weeks [29,32]. Following secondary syphilis, the disease progression enters a period referred to as the latent phase. Disease latency is characterized by a lack of clinical symptoms of syphilis, but serologic tests will still be positive. Tertiary syphilis is primarily associated with gummatous, cardiovascular, and neurological involvement. Tertiary syphilis is extremely rare and develops in the subset of untreated syphilis infections [31]. Gummatous syphilis can involve organs or supporting structures. It can result in infiltrative or destructive lesions leading to granulomatous lesions or ulcers (e.g., skin) or perforation/collapse of structure (e.g., palate, nasal septum), or organomegaly [32]. Classical late neurologic manifestations attributed to parenchymal damage include general paresis and tabes dorsalis [29]. Tabes dorsalis is the syphilitic involvement of the posterior columns of the spinal cord, which impacted about one-third of patients with late neurologic manifestations of syphilis in the pre-antibiotic era [29,32]. Figure 7 also illustrates the involvement of the cardiovascular system in tertiary syphilis, typically involving the ascending aorta, which in turn causes dilation of the aortic ring, aortic regurgitation, or ascending aneurysms [29,33]. Though not depicted in Figure 7, it is essential to understand that tertiary syphilis can occur following primary syphilis or secondary syphilis. As mentioned previously, the nonspecific symptoms noted with secondary syphilis mirror the clinical manifestation of monkeypox infection. Primarily, a disseminated maculopapular rash can be described in both monkeypox and syphilis infections [29]. Furthermore, the characteristic lymphadenopathy that can be noted in monkeypox is also a symptom of secondary syphilis [2,31]. Finally, a disseminated rash is visible in both monkeypox and secondary syphilis, as both may present with a diffuse maculopapular rash [29]. The disseminated rash of secondary syphilis most often, but not always, involves the palms and soles, though alternative anatomical locations have been noted [29,30]. Pale and discrete macular lesions initially appear on the trunk and proximal extremities; however, the number of lesions is intensely concentrated on the extremities [33]. While Cohen et al. specifically describe a disseminated rash on the scrotum in males, the disseminated rash associated with monkeypox is most noticed on the extremities and near the genitals. The cutaneous manifestations of secondary syphilis are diverse such that the rash can present as papular, annular, or pustular and have a fine overlying scale [29]. In monkeypox, the rash progresses through stages of macules, papules, vesicles, and pustules before it begins to scab and desquamate [2]. Secondary syphilis presents other cutaneous manifestations such as condylomata lata, mucous patches, or split papules [29]. These findings are specific to syphilis as no such similarities are found in monkeypox and, thus, become one of the clinically distinguishing features between monkeypox and syphilis. It is also imperative to understand that the rash of secondary syphilis is not pruritic and can be minimal enough to be overlooked. At the same time, lesions associated with monkeypox are often painful [34]. 

Smallpox

**Figure 8:**
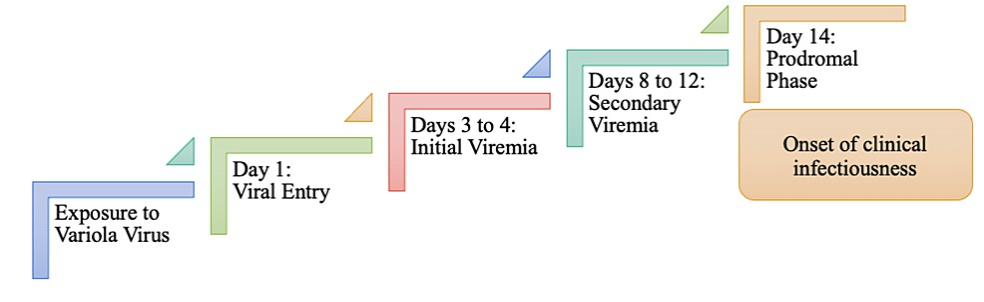
Sequential Progression of the Different Stages of Viral Replication Leading to Clinical Presentation of Smallpox

Though eradicated in the 1970s, smallpox has the most similarities to the clinical presentation of monkeypox compared to other similar infections. While monkeypox has been traced back to several different animal reservoirs, smallpox is a human disease without animal reservoirs, which became an essential factor in its successful eradication [2,35]. In the 20th century, before the eradication of smallpox, the global death toll of smallpox was well over 300 million [35]. Smallpox results from an infection of the variola virus, also known as the smallpox virus. The variola virus belongs to the *Orthopoxvirus* genus of the Poxviridae family [36,37]. Variola shares many basic features of other orthopoxviruses, such that it has a linear genome containing approximately 200 genes; those in the central region encode proteins involved in the replication or the virion structure. These virological facts are also applicable to the monkeypox virus [2,37]. Variola virus measures approximately 300 nm to 350 nm long [37]. Like all other poxviruses, variola virus has a linear, double-stranded. DNA genomes are unique because their genetic makeup encodes all the proteins necessary for replication, allowing them to replicate in the host cell cytoplasm [35]. Variola replicates in the cytoplasm of an infected cell and then invades the epithelium of the dermal layer [38]. Before the eradication of smallpox, exposure to the variola virus primarily occurred through direct contact. Like monkeypox, smallpox belongs to the Poxviridae family and, thus, shares similar pathogenesis. The viral entry of the variola virus occurs through the oropharynx or nasopharynx [2,35]. Once the virus enters, it will migrate to regional lymph nodes, where primary replication begins. Migration from regional lymph nodes to distal lymph nodes and lymphoid organs such as the bone marrow and the spleen constitutes the initial viremia, referred to in Figure 8. The virus enters systemic circulation from the lymphoid organs and lymph nodes and makes its way to target organs during secondary viremia. As noted in Figure 8, the period of clinical infectivity is referred to as the prodromal phase, when smallpox clinical manifestations begin to appear. Signs and symptoms may include initial nonspecific findings such as fever, chills, abdominal pain, vomiting, headache, and backache [35,39]. 

Like monkeypox, smallpox rash also progresses through various stages, with approximately 48 hours elapsing between the stages [35,36]. Clinical manifestation begins when small red lesions appear on the patient’s tongue and palate [39]. These oral lesions are referred to as enanthem. Eventually, these red spots will change into sores that will break open and spread the virus into the mouth and the throat [36]. Once the sores in the mouth have begun breaking, a rash begins to appear on the skin. The emergence of skin lesions begins on the forearms, face, and trunk and then spreads to the rest of the body, with palms and soles frequently [35]. Typically, the rash will spread to the rest of the body within 24 hours [36]. The stages of the rash in smallpox mirror the stages of monkeypox, as outlined in Figure 2. The rash will begin as a maculopapular rash that will turn into a vesiculopustular rash after 1-2 days. Vesicles will appear round and firm with dermal involvement and measure 2-5 mm in diameter [38]. Vesicles typically appear around four days after the maculopapular rash occurs, and around day 6, the vesicles will turn to pustules. The pustules are sharply raised, usually round and firm to touch, like peas under the skin [36]. Pustules will begin to crust and turn into scabs 9-10 days after the initial exposure to the virus [38]. Most scabs will have fallen off three weeks after the rash appears [36]. As with monkeypox, an individual with smallpox is also deemed contagious until all the scabs have fallen off. As the scabs fall off, an individual may be left with some scarring and areas of hyper- or hypopigmentation. As time progresses, these scars may become lighter and eventually disappear. Similar to monkeypox, severe complications of smallpox included potential ocular infection and subsequent blindness. Similarities in the clinical manifestations and the stages for the rash allow diagnosticians to understand how smallpox presents similarly to monkeypox. However, since its eradication, it is not a diagnosis of primary concern.

Rickettsialpox

Rickettsialpox is a mild, self-limited, zoonotic febrile illness caused by the agent *Rickettsia akari* (*R. akari*) spread by bites of infected mites [40]. Confirmed or suspected cases of rickettsialpox have been documented in at least 14 countries around the globe; however, most of the cases have been noted in New York City [41]. Usually, rickettsialpox is a self-limited febrile illness that may be confused with chickenpox [42]. Its confusion with chickenpox stems from the cutaneous manifestations and the clinical symptomatology. Unlike monkeypox, rickettsialpox does not have human-to-human transmission and, thus, has no sexual predilection. 

 Rickettsialpox is characterized by eschar formation at the location of a mite bite, followed by the onset of systemic symptoms and a more generalized papulovesicular rash [40]. *R. akari* is transmitted among house mice and several species of rodents [41]. The vector of *R. akari* is the colorless mite *Liponyssoides sanguineus* (*L. sanguineus*), which is typically found in mice and other rodents [40]. The bite of *L. sanguineus* is painless, and an individual may not be aware of the mite bite. Seven to ten days following the bite, a papulovesicular skin lesion with surrounding erythema may form at the location of the bite [40,42]. Three to seven days after the initial skin lesion develops, patients will present with systemic symptoms, including a high-grade fever, chills, headaches, and myalgias [40-41]. Subsequently, infected individuals will develop a sparse, generalized papulovesicular rash resembling chickenpox [40]. 

A generalized papulovesicular rash is also noted in monkeypox; however, the systemic symptoms in monkeypox can be noted before the presence of a rash in some individuals, whereas systemic symptoms are not noted in rickettsialpox until lesions have formed. However, due to the nonspecific systemic symptoms of rickettsialpox and monkeypox, World Health Organization (WHO) lists rickettsialpox infection as a potential differential diagnosis [1]. 

**Figure 9:**
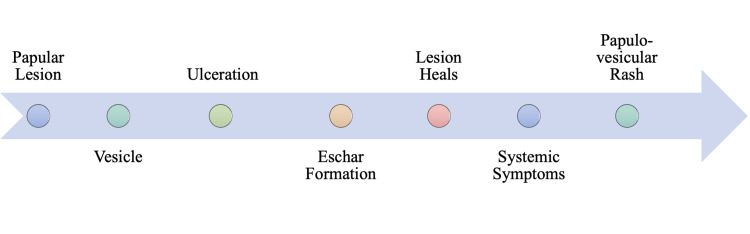
Proposed Timeline from Liponyssoides sanguineus Bite to Presentation of Rash and Symptoms

*R. akari* will proliferate locally in the epidermis at the site of the bite. Seven to ten days after the bite, a firm, red papule roughly 1-1.5 cm in diameter will appear [40]. A few days after the initial appearance, the papular lesion will begin to vesiculate with a surrounding area of erythema, as outlined in Figure 9. The vesicular lesion will begin to ulcerate, forming an eschar [40-41]. The eschar will eventually heal, and three to seven days after healing, an individual may present with several nonspecific systemic symptoms. A rickettsialpox infection may manifest as a sudden high fever, chills, sore throat, rigor, profuse sweating, myalgias, and anorexia [40-42]. Regional lymphadenopathy at the draining site of the eschar is common and generalized lymphadenopathy has been reported [40]. Two to three days after the onset of the systemic symptoms, a generalized papulovesicular rash of rickettsialpox will erupt. As with the monkeypox rash, the papulovesicular rash of rickettsialpox is accompanied by an oropharyngeal enanthem [2,40]. A rickettsialpox rash typically lasts only one week [40-42]. In both cases, scabs will form and eventually crust off. The generalized papulovesicular rash is usually scattered on the face, trunk, and extremities with no sequence of involvement [40]. The vesiculopustular rash of monkeypox, however, follows a pattern of centrifugal distribution [2]. Like monkeypox, rickettsialpox is also a self-limited disease; however, antibiotics and supportive therapy may be used to relieve an individual from other systemic symptoms [40]. 

Scabies

Deemed a differential diagnosis of monkeypox by WHO due to the nature of its rash, scabies results from an infestation of the skin by the human itch mite [7,43]. Scabies is caused by a mite called* Sarcoptes scabiei var hominis*, an obligate human parasite measuring 300-400 mm [44]. Human scabies is a contagious skin infestation common in underserved and overcrowded populations and transmitted by close skin-to-skin or sexual contact [45]. It has been estimated that a patient with conventional scabies needs 5-20 minutes of close contact to transfer the mites from one person to another [44]. At room temperature, the mite can only live away from human skin for a brief period, typically 24-36 hours. Due to its limited lifespan, transmission through indirect contact through bedding or clothing is not as common [46].

**Figure 10:**
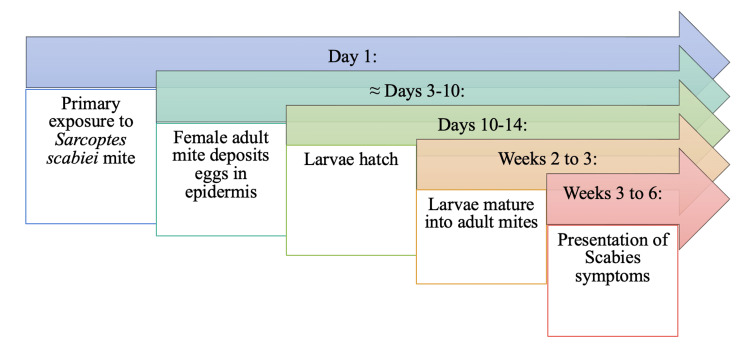
Proposed Timeline from Primary Exposure to Sarcoptes scabiei to Presentation of Symptoms

Because scabies results from an infestation with a mite, the proposed pathogenesis varies significantly from that of monkeypox, and the timeline of symptom progression is extended compared to monkeypox infection. A typical timeline of scabies pathophysiology is noted in Figure 10. Symptoms of scabies often begin one to four days after exposure [43]. A scabies-infested individual is considered contagious throughout the entire disease until they receive medical attention. Once an individual has been successfully treated for scabies and all mites and eggs have been destroyed, the individual is no longer considered contagious. The female mite burrows into the stratum corneum to lay eggs [45]. As the eggs hatch, they will leave the burrow and mature on the epidermis surface in adults [43,46]. The most common symptoms of scabies are produced by host immune reactions to burrowed mites and their byproducts [44]. It is essential to understand that the pathognomonic lesions of scabies infection are characterized by the mite burrow, which is seen as short, linear tracks ending with intact vesicles or erosions that contain the mite [46]. While the presence of the burrow is classical for scabies, it is only observed in a minute number of cases. Furthermore, the burrows are rarely visible to the naked eye and may not be noticed during a clinical examination.

The clinical presentation of scabies is defined by a slight erythematous papulovesicular rash, generally symmetrical, with a predilection for anterior axillary folds, the areolas, periumbilical skin, elbows, the volar surface of the wrists, interdigital web spaces, belt line, thighs, buttocks, penis, scrotum, and/or ankles without infection of the head and forehead in adults [44]. The erythematous papular or vesicular lesions noted in scabies are associated with the burrows [47]. As the characteristic rash of monkeypox is also a vesiculo-papular rash, scabies in these stages may be considered in a differential diagnosis. It is crucial to differentiate the physical manifestation of these conditions for proper diagnosing to get appropriate treatment and care. The primary means of treatment for scabies is through topical medications with or without oral treatment with ivermectin [43,47]. Unlike scabies, mild to moderate cases of monkeypox infection do not have a confirmed treatment plan. The focus is typically on supportive therapy to alleviate the discomfort of the symptoms. A scabies-infested individual is contagious until the individual has been medically treated; however, monkeypox is self-limited. An individual is considered contagious only until the last cutaneous lesion has crusted off and a new layer of skin has formed.

## Conclusions

Ensuring a correct diagnosis is crucial to providing appropriate treatments and care to an individual. As healthcare providers navigate through understanding the pathogenesis and clinical manifestations of monkeypox, understanding conditions that mimic its clinical manifestation is also of importance. The characteristic finding of monkeypox is the vesiculo-pustular rash; however, it is important to understand that secondary syphilis, rickettsialpox, scabies, molluscum contagiosum, varicella zoster, and smallpox all present with a similar rash. As the diagnosis of these disease states largely relies on clinical findings, physical examination and clinical history may be of the utmost importance in ensuring a correct diagnosis. Varicella zoster may be suspected based on childhood history and the distribution of a rash more commonly found on the trunk. Secondary syphilis, however, may be suspected based on a thorough clinical history that includes the sexual history of the individual, along with the location of the rash. Molluscum contagiosum and rickettsialpox are both diagnoses that may be suspected based on previous contact with a similar rash or history of tick bites or travel to areas with a high presence of ticks. Patient histories, including information about economic conditions, may provide insight into risk factors for a number of these infections, such as scabies. Eliminating suspicions of a different diagnosis further may allow for more appropriate use of diagnostic confirmatory tests and aid in expedient treatments. Furthermore, it allows for the ability to contact trace monkeypox cases while recommending isolation until the lesions are fully crusted, confirming the individual is no longer contagious. Appropriate treatment early on during the presentation of infection allows for a decreased risk of severe disease and a potential for increased quality of life following appropriate treatment and resolution of symptoms.
